# The Association of Muscle-Related Factors With Glaucoma and Related Traits in a Large United Kingdom Population

**DOI:** 10.1167/iovs.66.6.66

**Published:** 2025-06-23

**Authors:** Kian M. Madjedi, Kelsey V. Stuart, Grace S. Yin, Robert N. Luben, Zihan Sun, Mahantesh Biradar, Ruiqi Hu, Paul J. Foster, Peng T. Khaw, Katharina C. Bell, Jonathan G. Crowston, Anthony P. Khawaja, on behalf of the UK Biobank Eye and Vision Consortium

**Affiliations:** 1 NIHR Biomedical Research Centre, Moorfields Eye Hospital NHS Foundation Trust and UCL Institute of Ophthalmology, London, United Kingdom; 2 Ivey Eye Institute, Schulich School of Medicine, Western University, London ON, United Kingdom; 3 MRC Epidemiology Unit, University of Cambridge, Cambridge, United Kingdom; 4 NHMRC Clinical Trials Centre, University of Sydney, Camperdown, New South Wales, Australia; 5 Eye-ACP, Duke NUS Medical School, Singapore, Singapore; 6 Centre for Vision Research, Duke-NUS Medical School, Singapore, Singapore; 7 Singapore Eye Research Institute, Singapore, Singapore; 8 Save Sight Institute, Faculty of Medicine, University of Sydney, Camperdown, New South Wales, Australia

**Keywords:** glaucoma, intraocular pressure (IOP), neuroprotection, grip strength (GS), UK Biobank

## Abstract

**Purpose:**

The purpose of this study was to investigate the hypothesis that muscle-related factors influence glaucoma risk, we examined the association of grip strength (GS), thigh muscle volume (TMV), and walking pace (WP) with glaucoma and its related traits.

**Methods:**

We included UK Biobank participants with data on IOP (*N* = 114,284), optical coherence tomography (OCT) macular inner retinal layer thickness measures (*N* = 44,141) and glaucoma status (*N* = 105,556; 2006–2010). Linear regression was used to evaluate multivariable-adjusted associations of GS, TMV, and WP with IOP and macular inner retinal OCT parameters, and logistic regression was used to evaluate associations with glaucoma status. We additionally examined gene-GS interactions with each outcome using a polygenic risk score (PRS) that combined the effects of 2673 genetic variants associated with glaucoma.

**Results:**

After adjustment for key anthropometric, lifestyle, and medical covariables, we found each additional standard deviation (SD) increase in GS (8.6 kg in men and 6.1 kg in women) was associated with thicker macular retinal nerve fiber layer (mRNFL) by 0.08 µm (*P* = 0.013) and 0.07 µm (*P* = 0.010) in men and women, respectively; thicker macular ganglion cell-inner plexiform layer (mGCIPL) by 0.12 µm (*P* = 0.003) and 0.17 µm (*P* < 0.001); higher IOP by 0.15 millimeters of mercury (mm Hg; *P* < 0.001) and 0.16 mm Hg (*P* < 0.001) and lower odds of glaucoma (odds ratio [OR] = 0.83, *P* < 0.001) in men only. The association with glaucoma was replicated in the independent EPIC-Norfolk cohort. Faster WP and greater TMV were also associated with lower odds of glaucoma in men only (*P* = 0.004 and *P* = 0.017, respectively). Stronger GS-IOP associations were observed in participants with a higher level of genetic risk for glaucoma (P_interaction_ < 0.001).

**Conclusions:**

In this cross-sectional and gene-environment interaction study, factors relating to muscle strength, mass, and function were consistently associated with higher IOP, thicker inner retinal OCT measures in both sexes, and lower odds of glaucoma in men.

Physical activity has been linked to neuroprotective benefits in age-related neurodegenerative conditions, including Alzheimer's disease and Parkinson's disease.^1^^–^^4^ Exercise has also been shown to have a protective effect on retinal ganglion cells (RGCs) in rodents,^5^ reduce glaucoma risk in humans,^6^ as well as delay glaucoma progression both epidemiologically^7^^,^^8^ and in experimental models.^9^ Keen interest exists in the potential for neuroprotection in conditions like glaucoma, a disease marked by progressive RGC death, leading to irreversible vision loss. Although the precise mechanisms by which exercise protects RGCs remains unclear, growing evidence indicates that the broad effects of exercise on various organ systems may be mediated by myokines – factors released by contracting skeletal muscle, which have been shown to alter metabolic processes in other organs, including the eyes.^10^^,^^11^ Myokine signaling has been implicated in the upregulation of important neurotrophic factors (such as brain-derived neurotrophic factor [BDNF]) involved in neuronal survival, differentiation, and plasticity, and which have been associated with RGC survival and synapse formation in animal models.^12^^–^^19^ It remains unclear, however, whether skeletal muscle-related factors play a role in glaucoma and related traits (such as inner retinal thickness) in humans.

Easily collected biophysical parameters can directly and indirectly assess general skeletal muscle factors, foremost among which is hand grip strength (GS). This is widely regarded as a robust marker of overall muscle strength and is a noninvasive, objective, and easily collected clinical parameter. It is closely associated with muscle strength and contractility, and is a reliable predictor of cardiovascular disease, frailty, disability, and all-cause mortality.^20^^–^^25^ Walking pace (WP) has also been used an indicator of overall muscle function, physical fitness, and frailty, with self-reported pace being associated with cardiorespiratory fitness, and all-cause mortality.^25^ Fat-free thigh muscle volume (TMV), measured using magnetic resonance imaging (MRI), is another marker of muscle mass and has been used in assessing sarcopenia risk.^24^

We conducted a large, observational study evaluating the associations of GS (and secondarily, of the highly-related skeletal muscle factors WP and TMV) with glaucoma status and related traits, including intraocular pressure (IOP) and optical coherence tomography (OCT) derived measures of inner retinal thickness. Using data from the UK Biobank, with external validation in the EPIC-Norfolk Eye Study, we investigated the hypothesis that skeletal muscle-related factors influence glaucoma risk and/or related traits. Given evidence that some lifestyle risk factors may have a modifying effect only among those at higher genetic risk for glaucoma,^26^ we performed gene-environmental interaction analyses to investigate whether pre-existing genetic risk for glaucoma may modify any of these associations.

## UK Biobank

The UK Biobank is a large-scale, population-based cohort consisting of over half a million UK residents, aged 37 to 73 years at enrollment, registered with the National Health Service (NHS). Baseline data collection took place between 2006 and 2010 at 22 assessment centers. Participants completed detailed touch-screen questionnaires covering sociodemographic details, medical history, lifestyle factors, and various physical measurements.^27^ Multiple follow-up and supplementary assessments, including an eye and vision substudy conducted in 2009 to 2010, were carried out on subsets of participants.^28^ The UK Biobank study received approval from the National Health Service North West Multicentre Research Ethics Committee (06/MRE08/65) and the National Information Governance Board for Health and Social Care. This research was conducted under UK Biobank application number 36741 and adhered to the principles of the Declaration of Helsinki. All participants provided written informed consent prior to enrollment. The overall study protocol and specific protocols for individual tests are accessible online (https://biobank.ndph.ox.ac.uk/ukb/index.cgi).

### Assessment of Muscle-Related Factors

Whereas GS is the most commonly used and clinically relevant biomarker for muscle-related factors, WP and TMV are closely related markers of muscle strength, mass, and function. During the baseline assessment, hand grip strength was measured using a Jamar J00105 hand dynamometer, which assesses grip force isometrically and can be adjusted for hand size in 5.5-inch increments. Participants were instructed to sit upright with their arms resting on the armrests. The elbow of the arm holding the dynamometer was positioned against their side and bent at a 90-degree angle. Participants were asked to squeeze the handle of the dynamometer as forcefully as possible for 3 seconds with verbal encouragement. The dynamometer features a dual-scale readout that measures isometric grip force from 0 to 90 kg and includes a “peak hold” needle that displays the grip strength score upon release. Measurements from both the right and left hands of each participant were recorded. Prior studies using data from the UK Biobank have found the use of hand grip strength to predict health-related outcomes is comparable whether measured in absolute or relative terms. As a result, it was recommended that absolute units (i.e. per kg) be used, and therefore the mean of the GS measures from both hands was calculated and assessed in absolute units.^29^^,^^30^

WP was assessed with a self-reported single item question “how would you describe your usual walking pace?” with the participant’s options limited to “slow pace,” “steady/average pace,” and “brisk pace.” Fat-free TMV was assessed in approximately 40,000 UK Biobank participants using quantitative MRI with dual energy x-ray absorptiometry (DXA).

The MRI scans were part of a comprehensive imaging protocol within the UK Biobank. Participants eligible for inclusion were aged between 40 and 69 years. Individuals were excluded if they had metal or electrical implants, had undergone surgery within 6 weeks prior to scanning, or had medical conditions that contraindicated an MRI scan. All scans were performed using a Siemens Aera 1.5 T scanner (Syngo MR D13, Siemens, Germany). Fat-free TMV was defined as the volume of all voxels in the thigh with fat fraction of less than 50%, representing “viable muscle tissue.”

### Assessment of Glaucoma-Related Outcome Measures

The UK Biobank eye and vision substudy, introduced between 2009 and 2010, generated additional ophthalmic data for approximately 115,000 UK Biobank participants at 6 assessment centers. Detailed descriptions of the data collection methods for this study have been previously published.^28^ Briefly, IOP was measured in both eyes of approximately 115,000 participants using an Ocular Response Analyzer (ORA) non-contact pneumotonometer (Reichert Corp., USA), which generates an IOP that is least affected by biomechanical properties of the cornea (i.e. “corneal-compensated IOP”).^31^ To estimate pretreatment IOP for participants on IOP-lowering medication, we imputed pretreatment IOP by dividing the measured IOP by 0.7, a method described previously.^32^ The participant-level IOP was calculated as the average of measurements from both eyes, or from either the right or left eye if data from only one eye were available.

Approximately 65,000 participants had macular spectral domain OCT (SD-OCT) imaging during their baseline examinations.^28^ We used the mean thickness measurements of the macular retinal nerve fiber layer (mRNFL) and the macular ganglion cell-inner plexiform layer (mGCIPL; in µm) at the participant level in our analyses. It is important to note that peripapillary RNFL thickness measurements were not available in the UK Biobank.

Glaucoma status was assessed in approximately 175,000 participants using self-reported data from touchscreen questionnaires. We additionally included participants who had an International Classification of Diseases (ICD) code for glaucoma (ICD Ninth revision: 365.* (excluding 365.0); ICD Tenth revision: H40.* (excluding H40.0 and H42.*) in their linked hospital records at any time before, and up to 1 year after, the baseline assessment. We excluded individuals diagnosed with glaucoma prior to the age of 30 years, as well as healthy controls who reported using ocular hypotensive medication or carrying an ICD code for glaucoma suspect (ICD Ninth revision: 365.0 and ICD Tenth revision: H40.0), as described in previous publications.^32^^,^^33^

## EPIC-Norfolk Eye Study

We performed supplementary analyses using data from the EPIC Norfolk Eye Study for available outcome variables (including IOP and glaucoma status) for external validation of our primary analyses. EPIC-Norfolk is a UK branch of a larger European prospective cohort study; comprehensive methods have been detailed in other publications.^34^ Briefly, approximately 25,000 participants between 40 and 79 years of age were recruited from general practices across the region and were examined between 1993 and 1997. The third health examination (between 2004 and 2011) included a detailed ophthalmic assessment, with additional cross-sectional data collected on 8623 participants.^35^^,^^36^ Participants’ demographic information was collected, general health questionnaires covering lifestyle and diet were conducted, and participants each underwent a health check in which GS was measured using a hand-held Smedley Dynamometer (Scandidact, Kvistgaard, Denmark). The GS was recorded as the strongest force generated (in kilograms [kg]) achieved from 2 measurements per hand, while standing with their forearms bent at 90 degrees. Scanning laser polarimetry (GDx VCC) was used to measure average peripapillary RNFL thickness and IOPcc was measured using the ORA. Glaucoma status was assessed by an ophthalmic examination including visual acuity, tonometry, and evaluation of the optic nerve and peripapillary RNFL. Additional information about the assessment and diagnostic protocol is available in other publications.^36^ EPIC-Norfolk adhered to the principles of the Declaration of Helsinki and the Research Governance Framework for Health and Social Care. The study received approval by the Norfolk Local Research Ethics Committee (05/Q0101/191) and East Norfolk & Waveney NHS Research Governance Committee (2005EC07L). All participants provided written informed consent.

## Genotyping Data, Glaucoma Multi-Trait Analysis of Genome-Wide Association Study Polygenic Risk Score

For glaucoma-related genetic variants, we constructed a polygenic risk score (PRS) based on 2673 independent single nucleotide polymorphisms (SNPs) associated with glaucoma (*P* ≤ 0.001) from a recent multi-trait analysis (MTAG) of genome-wide association study (GWAS), which included the UK Biobank.^37^ Given the complexity of glaucoma as a disease, we considered the MTAG PRS to be a better representation of genetic variation in glaucoma than any individual or limited set of genetic variants. The effect estimates from the original MTAG study were then used to generate a glaucoma PRS for each individual participant, using a standard weighted sum of individual SNPs:

$$\sum_{i=1}^{2,673}{\hat{\beta }}_{\left(i\right)}*SNP_{\left(i\right)}$$

where the estimated effect size of *SNP*_(*i*)_ on glaucoma is represented by ${\hat{\beta }}_{\left(i\right)}.\;$Standardization of the PRS was then performed using a mean of 0 and a standard deviation (SD) of 1 for all analyses. We evaluated whether the association between GS and various glaucoma-related traits were modified by the glaucoma MTAG PRS by testing the significance of an interaction term between GS and the genetic factor in the maximally adjusted regression models.

### Statistical Analyses

The baseline characteristics of each analytical subgroup were summarized and reported as mean (SD) for continuous variables, and frequency (%) for categorical variables. To examine the key associations between muscle-related factors and glaucoma-related outcomes, we used multivariable linear regression for continuous outcomes (IOP, mRNFL, and mGCIPL thickness) and logistic regression for glaucoma status. We adjusted each of our analyses for key covariables, based on established risk factors for glaucoma and potential associations with muscle-related factors. The covariables included age (years), sex (female or male), self-reported ethnicity (White or non-White), social deprivation (using the Townsend Deprivation Index, which provides a measure of material deprivation based on postal code where higher scores reflect greater relative deprivation), height (cm), body mass index (BMI; calculated as weight/height^2^), systolic blood pressure (SBP; millimeters of mercury [mm Hg]; calculated as the average of 2 measurements), smoking status (never, current, or former), alcohol intake (infrequent, regular, former, and never use),^32^ as well as self-reported diabetes status (yes/no) and spherical equivalent (diopters [D]; calculated as spherical power + one-half cylindrical power using the average of available right and left eye measurements). Comprehensive details on the acquisition and measurement of each of these variables are available online (https://www.ukbiobank.ac.uk). As muscle-related factors are well-known to have different sex-specific distributions,^38^ we conducted all analyses stratified by sex. We also performed analyses adjusting additionally for age-squared to account for residual confounding by age.

## Results

**Figure 1.**
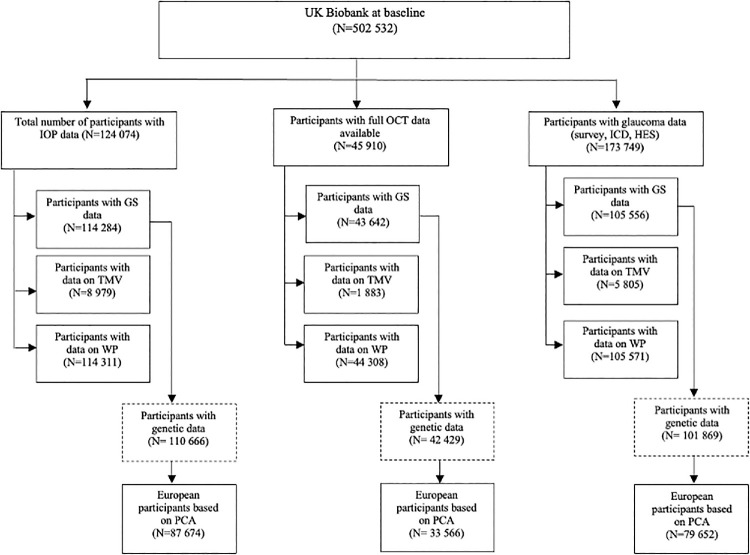
Flow diagram outlining eligible UK Biobank participants available for this study. GS, grip strength, HES, hospital episode statistics; ICD, international classification of disease; IOP, intraocular pressure; OCT, optical coherence tomography; PCA principal components analysis; TMV, thigh muscle volume; WP, walking pace.

We included 114,284 participants for the analysis of IOP, 43,642 participants for OCT-derived macular inner retinal thickness measures, and 105,556 participants for glaucoma status (Fig. 1). Table 1 summarizes the participant characteristics for each of these three analytical subgroups. Due to significant overlap among these groups, the demographic features and baseline characteristics were largely similar. As with the UK Biobank cohort in general, the mean age was 56.5 years; women comprised slightly over half of our participants (approximately 53%) and a majority of the participants were White (90%–91%). As in other studies,^38^ the distribution of GS in the UK Biobank was significantly different between men (38.5 kg, 8.7) and women (22.9 kg, 6.1) and, as such, all analyses were performed stratifying by sex. All analyses with continuous measures (i.e. IOP, mRNFL, and mGCIPL) were assessed using continuous measures, but to showcase overall relationships, data for these associations were presented per quartile and per SD change.

**Table 1.** Participant Characteristics by Cohort

	Analysis of IOP	Analysis of OCT Parameters	Analysis of Glaucoma Status
Sample size, *N*	114,284	43,642	105,556
Age, y, mean (SD)	56.7 (8.0)	56.4 (8.1)	56.8 (8.1)
Sex, *n* (%)			
Men	52,875 (46.3)	20,483 (46.9)	48,426 (45.9)
Women	61,409 (53.7)	23,159 (53.1)	57,130 (54.1)
Ethnicity, *n* (%)			
White	103,921 (90.9)	39,801 (91.2)	95,154 (90.2)
Non-White	10,363 (9.1)	3,841 (8.8)	10,402 (9.9)
Townsend deprivation index, mean (SD)	−1.1 (3.0)	−1.05 (3.0)	−0.99 (3.0)
Body mass index, kg/m^2^, mean (SD)	27.3 (4.5)	27.3 (4.5)	27.3 (4.5)
Height, cm, mean (SD)	168.7 (9.2)	169.0 (9.2)	168.6 (9.2)
Systolic blood pressure, mm Hg, mean (SD)	137.3 (18.3)	137.0 (18.3)	137.3 (18.3)
Smoking status, *N* (%)			
Never	63,823 (55.9)	23,940 (54.8)	58,616 (55.5)
Former	39,528 (34.6)	15,369 (35.2)	36,454 (34.5)
Current	10,933 (9.6)	4,333 (9.9)	10,486 (9.9)
Alcohol status, *N* (%)			
Never	5,567 (4.9)	1,967 (4.5)	5,438 (5.2)
Infrequent	13,785 (12.1)	5,238 (12.0)	13,176 (12.5)
Former	3,966 (3.5)	1,559 (3.6)	3,802 (3.6)
Regular	90,966 (79.6)	34,878 (79.9)	83,140 (78.8)
Diabetes status, *N* (%)			
No	108,211 (94.7)	41,480 (95.1)	99,688 (94.4)
Yes	6,073 (5.3)	2,162 (5.0)	5,868 (5.6)
Spherical equivalent, D, mean (SD)	−0.3 (2.7)	0.01 (1.9)	−0.34 (2.7)
Intraocular pressure, mm Hg, mean (SD)	16.1 (3.3)	15.9 (3.2)	16.0 (3.3)
Macular RNFL thickness, um, mean (SD)	28.9 (3.9)	28.9 (3.8)	28.9 (3.9)
Macular GCIPL thickness, um, mean (SD)	75.2 (5.2)	75.2 (5.2)	75.2 (5.2)
Glaucoma status, *N* (%)	1,482 (1.5)	576 (1.3)	1,890(1.8)
Mean grip strength			
Men	38.5 (8.7)	38.5 (8.6)	38.1(8.7)
Women	22.9 (6.1)	22.7 (6.0)	22.6 (6.1)
Mean thigh muscle volume			
Men	12.4 (1.7)	12.5 (1.7)	12.4 (1.7)
Women	8.3 (1.2)	8.4 (1.2)	8.3 (1.2)
Self-reported walking pace			
Men, *N* (%)			
Slow	3,607 (6.9)	1,309 (6.4)	3,506 (7.3)
Steady	27,499 (52.3)	10,629 (52.2)	25,342 (52.7)
Brisk	21,473 (40.8)	8,432 (41.4)	19,280 (40.1)
Women, *N* (%)			
Slow	4,454 (7.3)	1,640 (7.1)	4,372 (7.7)
Steady	32,387 (53.0)	12,384 (53.7)	30,280 (53.3)
Brisk	24,247 (39.7)	9,034 (39.2)	22,158 (39.0)

D, diopter; IOP, intraocular pressure; mGCIPL, macular ganglion cell-inner plexiform layer; mRNFL, macular retinal nerve fiber layer; SD, standard deviation.

Note: Analyses with thigh muscle volume and walking pace have a different number of participants than the overall analyses (number [*N*] for analyses with thigh muscle volume and IOP for men = 4378 and 4601 for women; for analyses with OCT parameters, *N* = 937; and 946 for men and women, respectively; for analyses with glaucoma *N* = 2787; and 3018 for men and women, respectively). Analyses of walking pace and IOP included 52,841 men and 61,470 women; WP and OCT parameters included 20,812 men and 23,496 women; WP and glaucoma status included 48,390 men and 57,181 women.

### Glaucoma Status

In examining the association between GS and glaucoma status, we included 48,426 men and 57,130 women with data on GS. We identified a significant association between GS and glaucoma status in men only: each additional SD increase in GS was associated with lower odds of glaucoma (odds ratio [OR] = 0.83, 95% confidence interval [CI] = 0.78–0.90, *P* < 0.001). A dose-response association in men was also identified: each additional increase in quartile of GS was associated with lower odds of glaucoma, with a significant trend (*P*_trend_ < 0.001; Table 2). Compared to men in the lowest quartile of GS, men in the highest quartile of GS had substantially lower odds of glaucoma (OR = 0.68, 95% CI = 0.55–0.85, *P* = 0.001). No association between GS and glaucoma status was identified in women. These sex-specific associations between GS and glaucoma status were replicated in supplementary analyses using the EPIC-Norfolk dataset, which found that higher GS was associated with lower odds of primary open-angle glaucoma in men only (OR = 0.77, 95% CI = 0.62–0.95, *P =* 0.014; Table 3).

**Table 2.** Association Between Grip Strength, Thigh Muscle Volume, and Walking Pace With Glaucoma Status

	Men		Women	
	OR (95% CI)	*P* Value	OR (95% CI)	*P* Value
Grip strength	*N* = 48,426		*N* = 57,130	
Per SD increase	0.83 (0.78 to 0.90)	**<0.001**	0.96 (0.88 to 1.03)	0.25
Quartiles				
Quartile 1	Ref		Ref	
Quartile 2	0.75 (0.65 to 0.89)		1.03 (0.87 to 1.22)	
Quartile 3	0.77 (0.65 to 0.91)		0.88 (0.72 to 1.07)	
Quartile 4	0.68 (0.55 to 0.85)		0.92 (0.73 to 1.15)	
*P*_trend_		**<0.001**		0.22
Thigh muscle volume	*N* = 2,787		*N* = 3,018	
Per SD increase	0.61 (0.41 to 0.92)	**0.017**	0.92 (0.56 to 1.50)	0.73
Quartiles				
Quartile 1	Ref		Ref	
Quartile 2	0.67 (0.33 to 1.35)		1.45 (0.56 to 3.72)	
Quartile 3	0.69 (0.31 to 1.52)		1.51 (0.54 to 4.19)	
Quartile 4	0.24 (0.08 to 0.79)		0.81 (0.21 to 3.07)	
*P*_trend_		**0.038**		0.90
Walking pace	*N* = 48,390		*N* = 57,181	
Slow	Ref		Ref	
Steady	0.74 (0.60 to 0.91)	**0.004**	0.75 (0.60 to 0.94)	**0.010**
Fast	0.64 (0.51 to 0.81)	**<0.001**	0.83 (0.65 to 1.06)	0.137

CI, confidence interval; OR, odds ratio.

Note: All models are adjusted for age, age squared, ethnicity, Townsend deprivation index; body mass index; diabetes mellitus status; systolic blood pressure; alcohol status; smoking status; height; spherical equivalent. One standard deviation in grip strength is equivalent to 8.7 kg in men and 6.1 kg in women.

For men, mean grip strength quartile 1 ranges from 1 to 34. Quartile 2 is 34.5 to 39.5, quartile 3 is 40 to 45, and quartile 4 is 45.5 to 85. For women, mean grip strength: quartile 1 is 1 to 19, quartile 2 is 19.5 to 23, quartile 3 is 23.5 to 27.5, and quartile 4 is 28 to 57. For men, TMV values for quartile 1 range from 5.1 to 11.2; quartile 2 from 11.2 to 11.2 to 12.3; quartile 3 from 12.3 to 13.5; and quartile 4 from 13.5 to 21.4. For women, the ranges for each quartile of TMV are as follows: quartile 1 ranges from 3.8 to 7.4; quartile 2 from 7.4 to 8.1; quartile 3 from 8.1 to 8.9; quartile 4 from 8.9 to 15.7.

Bold face entries indicate *P* values < 0.05.

**Table 3.** The Association of Mean Grip Strength With IOP, Peripapillary RNFL, and POAG Status in EPIC Norfolk

	IOP, mm Hg (*N*_men_ = 3,099, *N*_women_ = 3,703)		Peripapillary RNFL, µm (*N*_men_ = 937; *N*_women_ = 946)		Primary Open-Angle Glaucoma Status (*N*_men_ = 931; *N*_women_ = 942)	
	*β* (95% CI)	*P* Value	*β* (95% CI)	*P* Value	*OR* (95% CI)	*P* Value
Men						
Per SD increase	−0.00 (−0.17 to 0.17)	0.98	0.001 (−0.001 to 0.004)	0.19	0.77 (0.62 to 0.95)	**0.014**
Quartiles						
Quartile 1	Ref		Ref		Ref	
Quartile 2	0.17 (−0.24 to 0.57)		−0.004 (−0.009 to 0.002)		0.99 (0.64 to 1.53)	
Quartile 3	0.002 (−0.42 to 0.42)		−0.002 (−0.008 to 0.003)		0.68 (0.41 to 1.13)	
Quartile 4	−0.07 (−0.52 to 0.38)		0.003 (−0.002 to 0.009)		0.32 (0.16 to 0.67)	
*P _trend_*		0.56		0.20		**0.002**
Women						
Per SD Increase	0.25 (0.12 to 0.38)	**<0.001**	0.002 (0.0001 to 0.004)	**0.041**	1.23 (0.96 to 1.57)	0.10
Quartiles						
Quartile 1	Ref		Ref		Ref	
Quartile 2	0.14 (−0.19 to 0.48)		0.002 (−0.003 to 0.007)		1.03 (0.57 to 1.88)	
Quartile 3	0.13 (−0.21 to 0.47)		0.01 (0.002 to 0.012)		1.36 (0.74 to 2.48)	
Quartile 4	0.60 (0.25 to 0.96)		0.01 (0.002 to 0.012)		1.40 (0.72 to 2.74)	
*P _trend_*		**0.001**		**0.01**		0.23

β, beta coefficient; Q, quartile; RNFL, retinal nerve fiber layer.

Notes: All models adjusted for age; age squared; ethnicity, Townsend deprivation index; body mass index; diabetes mellitus status; systolic blood pressure; alcohol status; smoking status; height; spherical equivalent.

For men: Quartile 1 ranges from 1 to 30.5; Quartile 2 from 30.6 to 36, Quartile 3 from 36.5 to 41, and Quartile 4 from 41.5 to 68. For women, Quartile 1 ranges from 1 to 18.5; Quartile 2 from 19 to 22, Quartile 3 from 22.5 to 25.5 and Quartile 4 from 26 to 75.5.

Bold face entries indicate *P* values < 0.05.

We included 5805 men and 3018 women in our analyses with total fat-free TMV and glaucoma status and identified a significant association between greater TMV and lower odds of glaucoma in men only. For each SD increase in TMV in men, the odds of glaucoma were 0.61 times lower (OR = 0.61, 95% CI = 0.41–0.92, *P =* 0.017). Compared with men in the lowest quartile of TMV, those in the highest quartile had a substantially lower odds of glaucoma (OR = 0.24, 95% CI = 0.08–0.79, *P* = 0.016) with a significant trend (*P_t_*_rend_ = 0.038) across quartiles. These associations were not replicated in women. We included 48,390 men and 57,181 women in our analyses of WP with glaucoma status. Men who reported “steady” or “brisk” WP were found to have 0.74 and 0.64 times the odds of glaucoma (OR = 0.74, 95% CI = 0.60–0.91, *P =* 0.004 and OR = 0.64, 95% CI = 0.51–0.81, *P* < 0.001, respectively) compared with men reporting a “slow” WP. No clear association with WP and glaucoma was found in women.

### Inner Retinal Thickness

**Figure 2.**
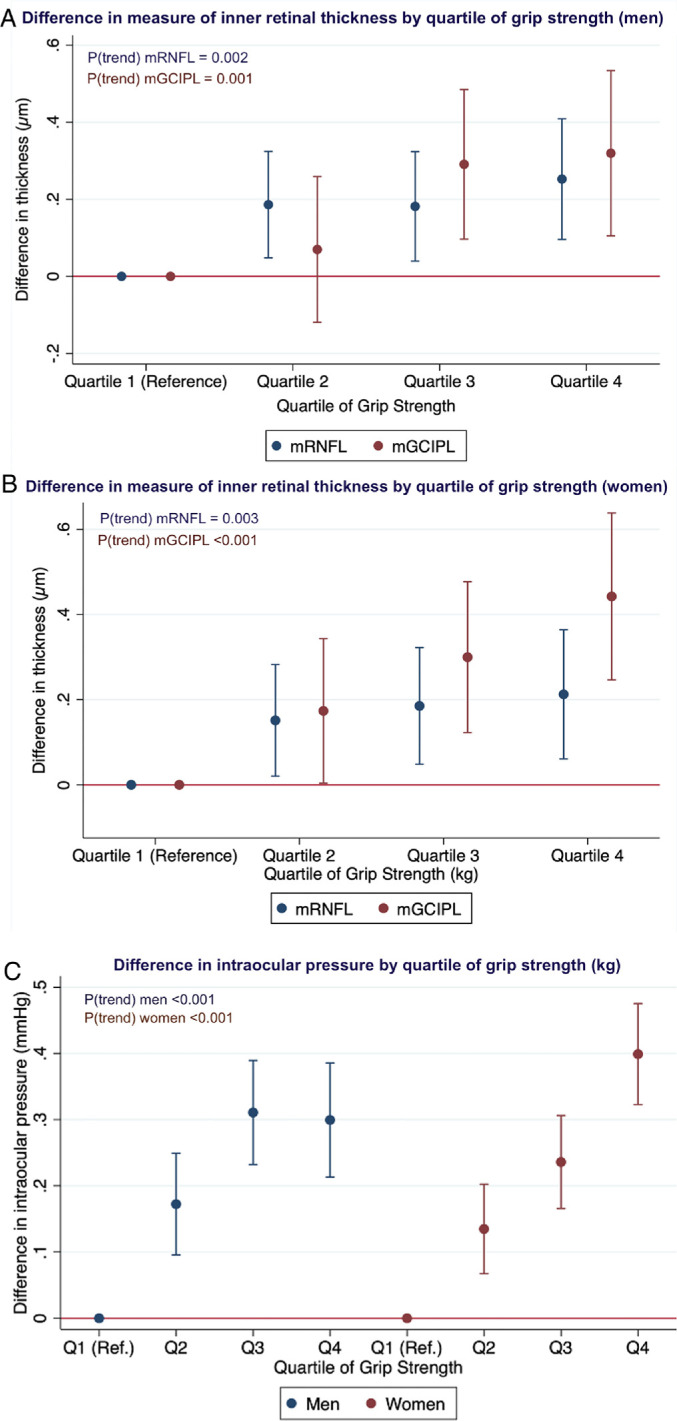
(**A**) Difference in measure of inner retinal thickness by quartile of grip strength in men. (**B**) Difference in measure of inner retinal thickness by quartile of grip strength in women. (**C**) Difference in intraocular pressure by quartile of grip strength in men and women.

We included 20,483 men and 23,159 women with data on mRNFL and mGCIPL thickness. Higher GS was associated with significantly thicker mRNFL and mGCIPL in both sexes. For each additional SD increase in GS, mRNFL was thicker by 0.08 µm in men and 0.07 µm in women, with a significant trend for higher mRNFL with increasing quartile of GS across both sexes (*P*_trend_ = 0.002 and 0.003 for men and women, respectively). Associations similar in magnitude and direction were also identified with mGCIPL: for each additional SD increase in GS, mean mGCIPL was thicker by 0.12 µm in men and 0.17 µm in women, with significant trends (*P*_trend_ = 0.001 and < 0.001 for men and women, respectively). Compared with those in the lowest quartile of GS, men in the highest quartile of GS had a thicker mGCIPL by 0.31 µm (95% CI = 0.10–0.52, *P* = 0.005) and women had a thicker mGCIPL by approximately half a micron (0.45 µm, 95% CI = 0.25–0.64, *P* < 0.001; Figs. 2A, 2B).

No association was found between TMV and mRNFL or mGCIPL in either sex, although these analyses were likely underpowered (Table 4). Faster WP was associated with a modestly thicker mRNFL in men only (Table 5). No association was found with WP and mGCIPL in either sex.

**Table 4.** Association of Fat-Free Thigh Muscle Volume With Intraocular Pressure and Optical Coherence Tomography Measures of Inner Retinal Thickness

	IOP, mm Hg (*N*_male_ = 4,378; *N*_female_ = 4,601)		mRNFL, µm (*N*_male_ = 937; *N*_female_ = 946)		mGCIPL, µm (*N*_male_ = 931; *N*_female_ = 942)	
	*β* (95% CI)	*P* Value	*β* (95% CI)	*P* Value	*β* (95% CI)	*P* Value
Men						
Per SD increase	0.01 (−0.15 to 0.16)	0.92	0.05 (−0.34 to 0.43)	0.81	0.00 (−0.47 to 0.48)	0.98
Quartiles						
Quartile 1	Ref		Ref		Ref	
Quartile 2	0.18 (−0.12 to 0.49)		0.18 (−0.60 to 0.96)		−0.05 (−0.99 to 0.90)	
Quartile 3	0.03 (−0.31 to 0.36)		0.24 (−0.61 to 1.10)		−0.20 (−1.25 to 0.86)	
Quartile 4	−0.06 (−0.46 to 0.34)		0.31 (−0.68 to 1.31)		−0.25 (−1.46 to 0.96)	
*P*_trend_		0.52		0.54		0.65
Women						
Per SD increase	−0.06 (−0.19 to 0.07)	0.34	−0.08 (−0.41 to 0.25)	0.65	0.33 (−0.13 to 0.79)	0.16
Quartiles						
Quartile 1	Ref		Ref		Ref	
Quartile 2	−0.06 (−0.33 to 0.21)		0.17 (−0.54 to 0.88)		0.65 (−0.32 to 1.63)	
Quartile 3	0.003 (−0.29 to 0.29)		0.21 (−0.55 to 0.97)		1.09 (0.04 to 2.14)	
Quartile 4	−0.20 (−0.54 to 0.13)		0.12 (−0.78 to 1.03)		0.83 (−0.42 to 2.07)	
*P*_trend_		0.34		0.79		0.15

Notes: All models adjusted for age; age squared; ethnicity, Townsend deprivation index; body mass index; diabetes mellitus status; systolic blood pressure; alcohol status; smoking status; height; spherical equivalent.

TMV: For men, quartile 1 ranges from 5.1 to 11.2; quartile 2 from 11.2 to 11.2 to 12.3; quartile 3 from 12.3 to 13.5; and quartile 4 from 13.5 to 21.4. For women, the ranges for each quartile of TMV are as follows: quartile 1 ranges from 3.8 to 7.4; quartile 2 from 7.4 to 8.1; quartile 3 from 8.1 to 8.9; and quartile 4 from 8.9 to 15.7.

**Table 5.** Association of Self-Reported Walking Pace With Intraocular Pressure and Optical Coherence Tomography Measures of Inner Retinal Thickness

	IOP, mm Hg (*N*_men_ = 52,841; *N*_women_ = 61,470)		mRNFL, µm (*N*_men_ = 20,182; *N*_women_ = 23,496)		mGCIPL, µm (*N*_men_ = 20,738; *N*_women_ = 23,478)	
	*β* (95% CI)	*P* Value	*β* (95% CI)	*P* Value	*β* (95% CI)	*P* Value
Men						
Slow	Ref		Ref		Ref	
Steady	0.31 (0.19 to 0.42)	**<0.001**	0.29 (0.07 to 0.50)	**0.009**	0.27 (−0.03 to 0.56)	0.07
Brisk	0.25 (0.13 to 0.37)	**<0.001**	0.32 (0.10 to 0.54)	**0.005**	0.29 (−0.02 to 0.60)	0.07
Women						
Slow	Ref		Ref		Ref	
Steady	0.15 (0.05 to 0.25)	**0.004**	0.02 (−0.18 to 0.22)	0.84	0.24 (−0.01 to 0.49)	0.06
Brisk	0.14 (0.03 to 0.24)	**0.011**	0.09 (−0.12 to 0.30)	0.40	0.23 (−0.04 to 0.50)	0.10

Notes: All models adjusted for age; age squared; ethnicity, Townsend deprivation index; body mass index; diabetes mellitus status; systolic blood pressure; alcohol status; smoking status; height; spherical equivalent.

Bold face entries indicate *P* values < 0.05.

In a supplementary analysis in EPIC, using data on peripapillary RNFL (ppRNFL), we identified a very modest association between greater GS and thicker ppRNFL, where each SD increase in GS was associated with a 0.002 µm thicker ppRNFL (see Table 3). These results were inconsistent and significant in women only.

### Intraocular Pressure

For analyses with IOP, we included 52,875 men and 61,409 women with data on GS and IOP. For each additional SD increase in GS, IOP was higher by approximately 0.15 mm Hg in both men and women (*P* < 0.001), with a significant trend for higher IOP with increasing quartile of GS (*P*_trend_ < 0.001 in both sexes; Fig. 2C). Faster WP was associated with a moderately higher IOP in both sexes, but no association with TMV was identified (see Tables 4, 5). The association of GS with IOP was additionally assessed in EPIC Norfolk, where an association between higher GS and higher IOP was significant in women only. Goldmann-correlated IOP was assessed as a sensitivity analysis, and the association with GS was only very slightly attenuated, although the direction and significance of the results were unchanged.

Sensitivity analyses were performed excluding (1) participants with glaucoma and (2) participants with IOP > 21 mm Hg. Across both sexes, all associations with GS, TMV, and WP remained unchanged in direction and significance, with only very modest attenuation in magnitude (attributable to modest loss of power in these analyses). Additional sensitivity analyses were performed without sex-stratification (i.e. including all participants – men and women) to assess for interaction with sex. These analyses identified a significant interaction with sex for GS (*P* for interaction = 0.009), TMV (*P* for interaction = 0.007), and WP (*P* for interaction = 0.04).

### Gene-Environment Interaction Analyses

**Figure 3.**
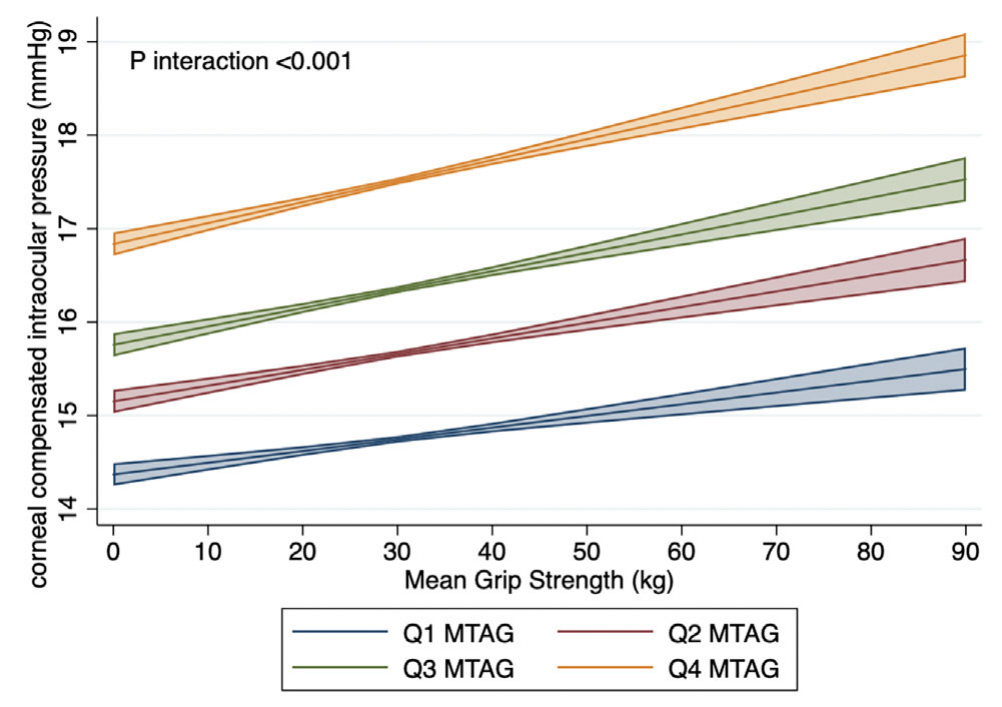
Gene-environment interaction analysis for the effect of the glaucoma MTAG PRS on the association between mean grip strength and intraocular pressure in participants of European ancestry.

We examined whether the association of GS with each of our outcomes of interest differs based on pre-existing genetic risk for glaucoma. We restricted these analyses to European participants based on genetic principal components analysis. We included 79,652 participants with data on glaucoma status, 33,566 with data on mRNFL, 33,477 with data on mGCIPL thickness, and 87,674 participants with data on IOP. The glaucoma MTAG PRS significantly modified the association between GS and IOP (*P*_interaction_ < 0.001) but not glaucoma status (*P*_interaction_ = 0.98), mRNFL thickness (*P*_interaction_ = 0.84), or mGCIPL thickness (*P*_interaction_ = 0.36; Fig. 3). Whereas higher GS was associated with higher IOP across all levels of genetic risk, this association was most pronounced in participants at the greatest underlying genetic risk for glaucoma. For those in the highest MTAG PRS quartile, each SD increase in GS was associated with a 0.11 mm Hg (95% CI = 0.05–0.16) higher IOP, compared to 0.00 mm Hg (95% CI = −0.03 to 0.02), 0.03 mm Hg (95% CI = −0.02 to 0.09) and 0.06 mm Hg (95% CI = 0.01 to 0.12) for those in quartiles 1 to 3 of the MTAG PRS, respectively.

## Discussion

In this large study using data from the UK Biobank, we investigated the association of muscle-related factors (specifically GS, TMV, and WP) with prevalent glaucoma and glaucoma-related traits, using both observational and gene-environment interaction analyses. We identified strong and consistent associations of higher GS, faster WP, and greater TMV with lower odds of glaucoma in men – a finding that was also replicated in a large external validation cohort. We also identified that higher GS was associated with thicker OCT-derived inner retinal measures and higher IOP in both sexes. The association between GS and IOP was modified by a glaucoma PRS, with stronger associations identified in participants in the highest level of genetic susceptibility to glaucoma. Overall, our findings support the hypothesis that muscle-related factors may potentially be protective in glaucoma and that these associations may be sex-specific.

As early-stage glaucoma can impact inner retinal structures in the macula, OCT assessment of the macular region can be helpful in diagnosis. In our analyses, we examined the association with mRNFL and mGCIPL, two key macular measures, and found that higher GS was associated with greater thickness in both layers. We additionally identified a distinct dose-response association, with a higher GS consistently associated with thicker both mRNFL and mGCIPL in both sexes. Although ppRNFL was not assessed in the UK Biobank, supplementary analyses in EPIC identified that higher GS was associated with very modestly thicker ppRNFL. This association was inconsistent and reached significance in women only. Ultimately, this is likely to be a chance finding, particularly as the EPIC cohort is much smaller than the UK Biobank (<1000 participants with data for either sex), compared with related analyses for macular RNFL in the UK Biobank (>20,000 participants for each sex). Taken collectively, our findings support that GS may be associated with thicker OCT-derived inner retinal measures in the general population.

Animal studies have suggested a potentially neuroprotective role of skeletal muscle contractility on RGCs: mice exposed to high levels of exercise were found to have significantly improved RGC functional recovery following an acute elevation in IOP^9^ or optic nerve transection.^39^ This preservation in RGC function was critically dependent on brain derived neurotrophic factor (BNDF) signaling and was additionally associated with reduced loss of RGC cell bodies, preservation of the inner-plexiform layer and reduction in activation of microglia.^5^ These effects have been hypothesized to be mediated at least in part by a tissue “cross-talk” phenomenon initiated by the release of neurotrophic factors from contracting skeletal muscle.^5^^,^^13^^,^^40^^–^^43^ Certain myokines, such as leukemic inhibitory factor (a myokine belonging to the IL-6 family), have been shown to mediate neuroprotection in RGCs following an episode of acute ocular hypertension, suggesting these protective effects may occur even in the presence of elevated IOP.^19^ We identified an association between muscle-related factors and modestly higher IOP in both sexes, and this may potentially be related to associations between muscle factors and corneal biomechanical properties. The protective associations with inner retinal measurements and glaucoma status suggest that any potentially neuroprotective effect of muscle-related factors on glaucoma or related traits may be via an IOP-independent mechanism and that the potential beneficial associations outweigh the association with higher IOP.

Faster WP has been found to correlate with higher levels of overall fitness, lower incidence of cardiovascular disease, and is a strong predictor of all-cause mortality.^44^^–^^48^ Studies of patients with glaucoma have demonstrated that individuals with more advanced glaucoma have a significantly slower WP at baseline, and experience a sharper decline in their WP over time.^49^^,^^50^ Our finding that faster WP was associated with a lower odds of glaucoma only in men further supports our hypothesis that muscle-related factors, including indirect measures of muscle function, might indeed play a potentially protective role in glaucoma.

Fat-free TMV is an objective, imaging-based biomarker of muscle mass. Low muscle volume, particularly in the form of sarcopenia, has been associated with negative cardiovascular outcomes and greater mortality^51^^,^^52^ and skeletal muscle atrophy has been shown to influence myokine expression and secretion.^53^^,^^54^ Our finding that greater TMV was associated with lower odds of glaucoma in men (even after adjusting for both age and age squared) lends further support to the potential protective association of muscle-related factors with glaucoma.

The strong and consistent protective associations between all muscle-related factors and glaucoma in men specifically is of particular importance to consider. Proposed mechanisms for these sex-specific associations may relate to the greater proportion of muscle mass and greater absolute muscle strength in men compared with women at all ages,^55^ or sex-differences in the type and magnitude of exercise-responsive myokine release following a period of exercise. IL-6, a key neurotrophic cytokine upregulated during physical activity,^56^ is differentially regulated in both sexes following a period of exercise. Blood samples from half-marathon runners pre- and post-exercise found that transcript levels of IL-6 in men demonstrated significant increases in IL-6 compared with matched women^57^ and another study found women participants took approximately 4 times longer to reach peak IL-6 levels compared with men following an equal bout of exercise.^58^ Sex differences in the composition of muscle fibers may also partly explain our identified associations: men tend to have greater proportional areas of so-called “fast-twitch” muscle fibers (which shorten at faster velocities and are responsible for power and speed) compared with women who have greater proportions of “slow twitch” fibers (responsible for endurance activity).^59^

It is ultimately likely that the identified sex-specific associations with glaucoma status are the result of interplay between various factors rather than any single mechanism. Ultimately, sex differences in patterns of physical activity, hormone levels, and muscle composition could contribute to the protective associations we identified in men. Notably, neither of the two assessed cohorts had a high number of extreme athletes, and our findings do not necessarily support that extreme exercise is more beneficial. Our current findings suggest that aiming for American Heart Association recommended targets for physical activity for both men and women would be reasonable.^60^

This study had a number of key strengths. First, it involved a large-scale analysis that examined several different glaucoma-related traits in relation to an array of important muscle-related factors, including measures of muscle strength, mass, and function. We also accounted for a wide spectrum of demographic, anthropometric, and lifestyle factors in our analyses, enabling a highly powered investigation of the relationship between glaucoma and muscle-related factors while reducing the likelihood of residual confounding. By using multiple glaucoma-related outcomes, we were able to identify significant and potentially important associations with traits such as IOP and inner retinal thickness. We specifically used corneal-compensated IOP as the measure of IOP in our analyses, as it may better represent true IOP compared with other measures, and has been more strongly associated with glaucoma.^31^ Considering the growing evidence that some lifestyle factors may only have a modifying effect on those with higher genetic risk of glaucoma, we also conducted gene-environment interaction analyses to examine whether GS might have differential associations with glaucoma based on one's baseline level of genetic risk for glaucoma. Although our PRS was derived from the UK Biobank, this study was not meant to validate or quantify this relationship. We used summary metrics that represent glaucoma risk for our analyses in this study. The independence of both marginal and interaction effects in the models limits the risks of data overfitting these gene-environment interaction analyses.

We used a broad set of criteria to assess glaucoma status, which included self-reported diagnosis of glaucoma, or history of glaucoma surgery or laser, and additionally included participants with an ICD code for glaucoma, and linked hospital records. The external validity of our findings is further supported by the replication of our key findings in a separate, large-scale population-based data set (EPIC-Norfolk).

One limitation of our study is the use of self-reported data for certain variables, including WP and medication use and for a component of glaucoma status ascertainment. Data obtained through questionnaires may be influenced by recall errors, social desirability biases, or potential misclassification. Muscle-related factors can be influenced by a myriad of other factors, including baseline physical fitness level, frailty, and age. We attempted to account for this by adjusting all our analyses for age as well as age squared, which are related to frailty and to glaucoma status and muscle-related factors. Our study is additionally limited by its use of cross-sectional analysis in both UK Biobank and EPIC, limiting determination of potentially causal associations.

We made efforts to account for as many potential confounding factors as possible; however, the observed associations may still be influenced by unmeasured variables that link the exposures and outcomes in our study. Reverse causality must also be considered in the interpretation of our findings, particularly with analyses of WP, as it has been shown that individuals with more advanced glaucoma (i.e. worse visual fields) may have a slower WP at baseline.^49^^,^^50^ Last, our findings may not be fully generalizable to other populations or ethnic groups, as the majority of our study participants (in both cohorts) were of European descent. This does not necessarily affect the internal validity of our results, particularly as a sensitivity analysis limited to non-White participants reduced the analysis cohorts by approximately 85% and was underpowered.

Our study found that factors relating to greater muscle mass, strength, and function are associated with lower odds of glaucoma in men, and thicker inner retinal parameters and higher IOP in both sexes. These findings provide indirect evidence that skeletal muscle-related factors may be implicated in glaucoma risk and that these associations may be sex-specific. Importantly, as GS and muscle mass can be enhanced through targeted interventions, these factors may represent potentially novel modifiable risk factors and may have important implications in terms of targeted lifestyle recommendations for glaucoma.
